# Sequencing and analysis of the complete mitochondrial genome of *Rugosa emeljanovi* (Anura: Ranidae)

**DOI:** 10.1080/23802359.2017.1347841

**Published:** 2017-07-07

**Authors:** Wanli Liu, Jiuchen Tao, Heng Wang, Wenge Zhao, Peng Liu

**Affiliations:** College of Life Science and Technology, Harbin Normal University, Harbin, Heilongjiang, P.R. China

**Keywords:** Ranidae, mitogenome, *Rugosa emeljanovi*, phylogenetic tree

## Abstract

In this study, the complete mitogenome sequence of *Rugosa emeljanovi* (Anura: Ranidae) is first determined using long PCR. It is a circular molecule of 17,733 bp in length (GenBank accession no. KU641020). Similar to the typical mtDNA of amphibians, the complete mtDNA sequence of *R. emeljanovi* contained 2 rRNA genes (12S rRNA and 16S rRNA), 22 tRNA genes, 13 protein-coding genes (PCGs), and a control region (D-loop). The nucleotide composition was 28.1% A, 26.8% C, 14.8% G, and 30.3% T. Mitochondrial genome analyses based on the NJ method yield phylogenetic trees, including the 13 already reported Ranidae family frogs. The molecular data presented here provide a useful tool for systematic analyses of genus *Rugosa* and *Glandirana*.

The rough-skinned frog (*Rugosa emeljanovi*) found in Northeast China is a small frog, belonging to the genus *Rugosa* of family Ranidae. This species is mainly found in the northeastern China (Heilongjiang, Jilin and Liaoning), Russia (southern Primorye), and Korean Peninsula (Fei et al. 2010). Since it has been described by the specimen from Yimianpo, Shangzhi County, Heilongjiang Province, China, the species is not found in this province for a long time (Zhao et al. 2008). The morphological characteristics of *R. emeljanovi* are similar to other sympatric amphibian species, including the frog *Rana nigromaculata* and even the toad *Bufo gargarizans*. So, molecular marker can provide a useful tool in studying species classification, origin, and evolutionary history (Lee et al. 1999; Zhang et al. 2005; Crimi and Rigolio 2008; Li and Yang 2015). In this paper, the complete mitochondrial genome of *R. emeljanovi* is sequenced for the first time with a muscle sample using a primer walking strategy and the long and accurate PCR. The specimen was collected from Jinlin Province of China (42°36'50.3″N, 127°50′59.5″E) and was stored in Zoological and Botanical Specimen Museum of Harbin Normal University (its accession number is HRB1506079).

The complete mtDNA of *R. emeljanovi* is 17,733 bp in length and contains 2 rRNA genes (12S rRNA and 16S rRNA), 22 tRNA genes, 13 protein-coding genes (PCGs), and a control region (D-loop). The nucleotide composition is 28.1% A, 26.8% C, 14.8% G, and 30.3% T. The accurate annotated mitochondrial genome sequence was submitted to GenBank with accession number KU641020.

Within the mitochondrial genome of *R. emeljanovi*, there are 8 reading frame overlaps (share 1–10 nucleotides) and 9 intergenic spacers (range from 1 to 31 bp). Except for *ND6* and 8 tRNA genes, all other mitochondrial genes were encoded on the heavy strand (H strand). In 13 protein-coding genes, except *ND2* with ATT, *COI* with ATA, *COIII* with TTA, the other 10 genes begin with ATG as start codon. *ND1*, *COII*, *ATP6*, *ND3* and *ND4* end with a single stop nucleotide T, *ND2*, *ATP8* and *CYTB* end with TAG, *COI* ends with AGG, *COIII* ends with CAT, *ND4L* ends with TAA, *ND5* ends with TCT, *ND6* ends with AGA. The 22 tRNA genes with the size ranging from 64 to 73 bp are interspersed along the whole genome. The sequence length of the 12S and 16S rRNA is 933 and 1584 bp, D-loop region is 2378 bp. In the WANCY cluster of tRNA genes, a 25 bp sequence is considered as the putative L strand replication origin (OL).

Mitochondrial genomes analyses based on MP, ML, and NJ yielded identical phylogenetic trees, including that 13 reported family Ranidae frogs (Figure 1). It appeared that *R. emeljanovi* and *Glandirana tientaiensis* formed a monophyletic group and they have a close genetic relationship. This study will facilitate the further research on compare the genetic structure of the related genus such as *Rugosa* and *Glandirana* and systematic analyses of family Ranidae (Sekiya et al. 2012; Xia et al. 2014; Shimada 2015).

**Figure 1.**
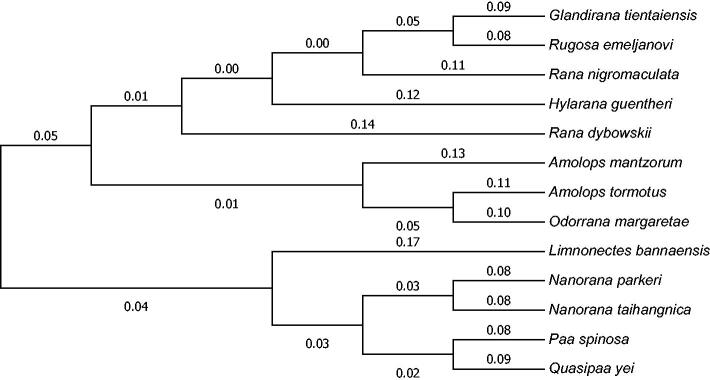
Phylogenetic tree generated using the Neighbor-Joining method based on complete mitochondrial genomes of some species in Anura: Ranidae. *Glandirana tientaiensis* (NC_025226), *Rugosa emeljanovi* (KU641020), *Rana nigromaculata* (NC_002805), *Hylarana guentheri* (NC_024748), *Rana dybowskii* (NC_023528), *Amolops mantzorum* (NC_024180), *Amolops tormotus* (DQ835616), *Odorrana margaretae* (NC_024603), *Limnonectes bannaensis* (NC_012837), *Nanorana parkeri* (NC_026789), *Nanorana taihangnica* (NC_024272), *Paa spinosa* (FJ432700), *Quasipaa yei* (NC_024843).
